# Prenatal exposures to mixtures of endocrine disrupting chemicals and children’s weight trajectory up to age 5.5 in the SELMA study

**DOI:** 10.1038/s41598-021-89846-5

**Published:** 2021-05-26

**Authors:** Katherine Svensson, Eva Tanner, Chris Gennings, Christian Lindh, Hannu Kiviranta, Sverre Wikström, Carl-Gustaf Bornehag

**Affiliations:** 1 Department of Health Sciences, Karlstad University, Universitetsgatan 2, 651 88 Karlstad, Sweden; 2 Department of Environmental Medicine and Public Health, Icahn School of Medicine at Mount Sinai, New York, NY USA; 3 Division of Occupational and Environmental Medicine, Lund University, Lund, Sweden; 4 Environmental Health Unit, Finnish Institute for Health and Welfare, Kuopio, Finland; 5 School of Medical Sciences, Örebro University, Örebro, Sweden

**Keywords:** Risk factors, Epidemiology, Paediatric research

## Abstract

Exposure to endocrine disrupting chemicals (EDCs) may impact early growth, although information is limited on exposure to combination of multiple EDCs. We aimed to evaluate the effect of prenatal exposure to EDC mixtures on birthweight z-scores and childhood weight trajectories. Twenty-six proven and suspected EDCs, were analyzed in prenatal urine and blood samples from 1118 mothers participating in the Swedish Environmental Longitudinal Mother and child Asthma and allergy (SELMA) study. Two growth parameters were estimated from each child’s weight trajectory from birth to 5.5 years of age: infant growth spurt rate and age at infant peak growth velocity (PGV). Weighted quantile sum (WQS) regression was used to estimate the mixture effect and identify chemicals of concern. A one-unit increase in the EDC mixture WQS index, was associated with decreased birthweight z-scores of 0.11 (95% CI − 0.16, − 0.06), slower infant growth spurt rate of 0.01 (95% CI − 0.03, − 0.01, on the log_10_ scale), and delayed age at infant PGV of 0.15 months (95% CI 0.07, 0.24) after adjusting for potential confounders. Stratified analysis by sex, showed that delayed age at infant PGV was mostly observed in girls with 0.51 months (95% CI 0.26, 0.76). Identified chemicals of concern included perfluorinated alkyl substances (PFAS), Triclosan, phthalates, non-phthalate plasticizers, bisphenols, polycyclic aromatic hydrocarbons, pesticides and PCBs. Prenatal exposure to EDC mixtures was associated with lower birthweight and altered infant weight gain trajectories.

## Introduction

Optimal fetal and infant growth are important to promote child development and prevent disease outcomes later in life^1–3^. It has been suggested that periods of faster weight gain in early infancy may be related to obesity in children and adults^4,5^. While early catch-up growth may improve neurodevelopment among low-birth-weight infants or infants born small-for-gestational age (SGA) it may come with a risk for future cardiovascular disease^6,7^. Fetal and infant growth is influenced by genetic, environmental, and social-behavioral factors^8^. The hypothesis of developmental origins of health and disease (DOHaD) indicates that environmental stressors during pregnancy may lead to later health effects both in childhood and adulthood^9^. Barker first showed evidence of the DOHaD hypothesis with his research linking poor maternal nutrition to lower birth weight and metabolic diseases later in life^10^. Thus, besides well-known factors, such as parental height or breastfeeding^8,11^, environmental factors may influence early growth and development of disease.

Of special concern are those chemicals with suspected or proven endocrine disrupting properties (EDCs) (e.g., phthalates, perfluorinated alkyl substances (PFAS), and polychlorinated biphenyls (PCBs)) which have been associated with adverse health effects impacting the metabolism (e.g., diabetes, insulin resistance), neurodevelopment (e.g., IQ), respiratory (e.g., asthma) and reproductive (e.g., early puberty) health^12,13^. EDCs are found in many daily used products (e.g., personal care products, pesticides, antibacterials)^12^, and even though some are rapidly metabolized in the human body, continuous exposure may lead to pseudo-persistence^13,14^. Other EDCs are persistent and remain in the environment for decades even after being banned from use, such as dichlorodiphenyltrichloroethane (DDT), PCBs, and perfluorooctane sulfonate (PFOS) which can still be found in wildlife, soil, and water sources^12,15^. Developing fetuses are considered especially vulnerable as EDCs may pass through the placenta^12,16^, and exposure to EDCs during sensitive periods of development may result in permanent damage with long-term health effects^17^.

In regards to children’s growth, prenatal exposure to PFASs and PCBs has been associated with lower birthweight^18–20^, as well as lower weight during the first year of life^21^. In the same way there is evidence that suggest prenatal exposure to certain phthalates and phenols may result in lower birthweight^22–25^. In addition, polycyclic aromatic hydrocarbons (PAHs) have also been associated with smaller birth size and lower weight during the first two years of life^26^. Diversely, prenatal exposure to organochlorine pesticides (e.g., DDT and hexachlorobenzene (HCB)) have been associated with faster growth rate in the first year of life^27,28^. Results from two studies suggest that in a mixture of different classes of EDCs, PFAS, organophosphate pesticides, and lead had the strongest association with lower birthweight^29^, and organochlorine pesticides with increased BMI z-scores in childhood^30^. Some of these studies have also shown sex-specific effects in birthweight and growth in early childhood^18,19,21,31^. Even though humans are exposed to many chemicals at the same time in complicated mixtures of EDCs, there is limited knowledge on how prenatal exposures to EDCs, especially their concurrent and combined mixture effect, may impact pre- and postnatal growth.

The mentioned studies have evaluated growth parameters (e.g. birthweight, BMI) at a few specific time points or change in BMI z-scores between two moments in time. This provides information on the change in growth parameters between two time points but says very little about how growth changes over longer periods of time. A modelling approach of children’s weight measured over time would provide a more detailed tool to assess exposure effects on weight trajectories and characteristics of growth (e.g. latency). Therefore, studies evaluating if exposure to EDC mixtures may alter children’s weight gain over time is warranted. Previous results from the SELMA study, using single-chemical analysis, has shown associations between PFOA and lower birthweight^19^ and altered weight trajectory^32^. In this study, we evaluated the effect of prenatal exposure to a mixture of 26 proven and suspect EDCs on children’s birthweight and weight trajectory parameters from birth until 5.5 years of age using data from 1118 women and their children participating in the *Swedish Environmental Longitudinal, Mother and child, Asthma and allergy* (SELMA) study. We also examined potential sex differences in the relationship between prenatal EDCs exposure and weight trajectories.

## Results

Women in our sample had a mean age of 31 ± 5 years and BMI of 25 ± 4 kg, and children’s mean birthweight was 3.6 ± 0.5 kg (Table 1). The infant growth spurt rate was 0.37 ± 0.2 kg/month and age at infant PGV was 3.42 ± 1.7 months, with boys having a faster growth spurt rate (0.38 ± 0.2 kg/month) and earlier infant age at PGV (3.22 ± 1.5 months) than girls (0.35 ± 0.2 kg/month and 3.63 ± 1.8 months, respectively) (p < 0.001). The urinary phthalate metabolites MEP, MBP and ΣDEHP had the highest geometric mean (GM) at 67.5, 67.4 and 63.8 ng/mL, respectively (Table 2). In serum, PFOS had the highest GM at 5.5 ng/mL followed by PFOA at 1.6 ng/mL, and in plasma the GM of the ΣPCBs was 0.4 ng/mL. The concentrations of the 26 compounds did not differ by sex (p-value > 0.05).

**Table 1:** Sociodemographic characteristics of the study population and children’s growth parameters, n = 1118.

	Overall (n = 1,118)	Boys (n = 584)	Girls (n = 534)	P-value*
Continuous variables	Mean (SD)	Mean (SD)	Mean (SD)	
Maternal age (years)	30.9 (4.7)	30.9 (4.5)	30.9 (4.8)	0.842
Maternal BMI (kg/m^2^)	24.7 (4.3)	24.6 (4.2)	24.7 (4.3)	0.836
Infant’s gestational age at birth (weeks)	39.5 (1.7)	39.4 (1.8)	39.5 (1.5)	0.310

Growth parameters	Mean (SD), [Min–Max]	Mean (SD), [Min–Max]	Mean (SD), [Min–Max]	
Birthweight (kg)	3.610 (0.543), [1.449–5.695]	3.653 (0.558), [1.449–5.480]	3.564 (0.523), [1.865–5.695]	0.006
Birthweight z-scores	− 0.08 (1.03), [− 3.99, 4.48]	− 0.13 (1.08), [− 3.99, 4.48]	− 0.02 (0.98), [− 2.70, 3.49]	0.061
Infant growth spurt rate (kg/months)	0.37 (0.17), [0.09–1.07]	0.38 (0.18), [0.10–1.07]	0.35 (0.17), [0.09–0.98]	< 0.001
Infant Age at PGV (months)	3.42 (1.65), [0.09–8.85]	3.22 (1.51), [0.13–8.25]	3.63 (1.76), [0.09–8.85]	< 0.001

Categorical variables	n (%)	n (%)	n (%)	
**Maternal Education**				
Primary school or high school	402 (36.0)	207 (35.4)	195 (36.5)	
College or higher	716 (64.0)	377 (64.6)	339 (63.5)	0.756
**Smoking**				
Non-smoker	1,056 (94.5)	554 (94.9)	502 (94.0)	
Smoker	62 (5.5)	30 (5.1)	32 (6.0)	0.622
**Parity**				
Nulliparous	544 (48.7)	279 (47.8)	265 (49.6)	
Multiparous	574 (51.3)	305 (52.2)	269 (50.4)	0.576
**Breastfeeding until 3 months**				
No	115 (12.3)	59 (12.1)	56 (12.6)	
Yes	817 (87.7)	430 (87.9)	387 (87.4)	0.867

PGV = Peak growth velocity.

^*^P-value from Student t-test for continuous variables and Chi-square test for categorical variables.

**Table 2:** Concentrations of 26 compounds (ng/mL) in prenatal urine (not creatine adjusted) and blood samples, overall and by sex, n = 1,118.

Components of the EDC mixture				Overall (n = 1,118)			Boys (n = 584)		Girls (n = 534)		P-value*	
Matrix	Chemical Type	Parent compound (if applicable**)**	Analyte	LOD/LOQ^a^	% ≥ LOD	GM (GSD)	GM (GSD)		GM (GSD)			
**Urine**	Phthalates	DEP	MEP	0.010	100	67.5 (2.9)	69.1 (2.8)		65.7 (3.1)		0.432	
		DBP	MBP	0.100	100	67.4 (2.2)	69.4 (2.2)		65.4 (2.2)		0.211	
		BBzP	MBzP	0.040	100	15.6 (2.9)	15.7 (2.8)		15.5 (3.0)		0.812	
		DEHP	ΣDEHP^b^	–		63.8 (2.4)	62.4 (2.3)		65.4 (2.5)		0.360	
		DINP	ΣDINP^c^	–		25.6 (3.0)	25.3 (3.0)		26.0 (3.0)		0.688	
		DiDP/DPHP	MHiDP	0.031	100	1.23 (2.8)	1.25 (2.7)		1.20 (2.8)		0.600	
			MCiNP	0.031	99.9	0.66 (2.4)	0.68 (2.4)		0.65 (2.5)		0.305	
	Plasticizer	DiNCH	MOiNCH	0.023	99.0	0.30 (4.0)	0.31 (4.1)		0.30 (4.0)		0.887	
		TTP	DPP	0.042	100	1.38 (2.6)	1.44 (2.6)		1.32 (2.5)		0.115	
	Antibacterial		Triclosan	0.100	92.4	1.34 (10.1)	1.36 (10.5)		1.31 (9.7)		0.762	
	Bisphenols		BPA	0.050	100	1.48 (2.4)	1.51 (2.3)		1.47 (2.4)		0.636	
			BPF	0.024	90.3	0.15 (5.2)	0.16 (5.1)		0.15 (5.3)		0.535	
			BPS	0.009	97.5	0.07 (2.9)	0.07 (2.8)		0.06 (3.0)		0.079	
	PAH		2OHPH	0.003	100	0.20 (2.3)	0.20 (2.3)		0.21 (2.3)		0.582	
	Pesticide	Chlorpyrifos	TCP	0.035	100	1.30 (2.5)	1.30 (2.5)		1.29 (2.6)		0.880	
		Pyrethroids	3-PBA	0.017	99.0	0.16 (2.8)	0.16 (2.7)		0.16 (2.8)		0.964	
**Serum**	PFAS		PFOA	0.020	100	1.63 (1.7)	1.65 (1.7)		1.61 (1.8)		0.451	
			PFOS	0.060	100	5.49 (1.7)	5.56 (1.7)		5.42 (1.7)		0.406	
			PFNA	0.010	100	0.55 (1.7)	0.54 (1.7)		0.55 (1.7)		0.769	
			PFDA	0.020	100	0.26 (1.6)	0.26 (1.6)		0.26 (1.6)		0.897	
			PFUnDA	0.020	99.7	0.22 (1.9)	0.22 (1.9)		0.22 (1.8)		0.751	
			PFHxS	0.030	100	1.31 (1.8)	1.32 (1.8)		1.29 (1.8)		0.530	
**Plasma**	Organo-chlorine pesticide		HCB	0.005	100	0.05 (1.4)	0.05 (1.4)		0.04 (1.4)		0.347	
			Trans-Nonachlor	0.005	77.5	0.01 (1.8)	0.01 (1.8)		0.01 (1.8)		0.989	
		DDT	ΣDDT/DDE^d^	–		0.20 (2.0)	0.21 (2.0)		0.20 (2.0)		0.166	
	PCB		ΣPCB^e^	–		0.36 (1.7)	0.37 (1.7)		0.36 (1.7)		0.500	

Abbreviations: GM = Geometric mean, GSD = Geometric standard deviation, LOD = limit of detection, LOQ = limit of quantification.

Notes: Values < LOD retained the machine read value for urine and serum compounds, values < LOQ were substituted with LOQ/ 2 for plasma compounds. Further description of each analyte can be found in supplementary table S1.

^a^ LOD reported for all urine and serum compounds, LOQ reported for plasma compounds.

^b^ Molar sum of metabolites: mono-2-ethylhexyl, mono(2-ethyl-5-hydroxyhexyl), mono(2-ethyl-5-oxohexyl), and mono(2-ethyl-5-carboxypentyl) phthalates.

^c^ Molar sum of metabolites: mono(hydroxyisononyl), mono(oxoisononyl), and mono(carboxyisooctyl) phthalates.

^d^ Sum of DDT and its metabolite dichlorodiphenyldichloroethylene.

^e^ Sum of PCB congeners 74, 99, 118, 138, 153, 156, 170, 180, 183, 187.

*P-value from Student t-test comparing metabolite concentrations on the log-scale by sex.

The WQS regression models showed that one-unit increase in the EDC mixture WQS indices in deciles (range 0–9), were associated with lower birthweight z-scores (Beta = − 0.11; 95% CI − 0.16, − 0.06), slower infant growth spurt rate (Beta = − 0.01; 95% CI − 0.03, − 0.01, on the log_10_ scale), and later age at infant PGV (Beta = 0.15; 95% CI: 0.07, 0.24) (Table 3). A one-unit increase in WQS index is associated with a decrease in growth rate with 0.01 units on the log scale. In this study population it would represent a slower growth rate of 0.87 kg/month for children with high WQS index (90th percentile) as compared to 0.93 kg/month for children with low WQS index (10th percentile). The stratified WQS model with interaction term included, showed significant differences in the WQS index estimate by sex for age at infant PGV, with later age for girls (Beta = 0.51; 95% CI: 0.26, 0.76), but not for boys (Beta = − 0.04; 95% CI: − 0.30, 0.22) (p-value for interaction = 0.002). This would represent a two week delay for girls in age at infant PGV. There were no significant differences by sex for birthweight z-scores or infant growth rate.

**Table 3:** Adjusted associations^†^ from the WQS linear regression between prenatal EDC mixture and children´s growth characteristics, overall and by sex, n = 1,118.

	Overall (n = 1,118)	Boys^‡^ (n = 584)	Girls^‡^ (n = 534)	p-value_int_
	WQS index estimate (95% CI), p-value	WQS index estimate (95% CI), p-value	WQS index estimate (95% CI), p-value	
Birthweight z-scores	− 0.11 (− 0.16, − 0.06), < 0.001	− 0.22 (− 0.37, − 0.07), 0.004	− 0.29 (− 0.44, − 0.14), < 0.001	0.526
Log_10_ of infant growth spurt rate (kg/months)	− 0.01 (− 0.03, − 0.004), 0.007	− 0.08 (− 0.11, − 0.05), < 0.001	− 0.05 (− 0.08, − 0.01), 0.005	0.103
Infant age at PGV (months)	0.15 (0.07, 0.24), < 0.001	− 0.04 (− 0.30, 0.22), 0.760	0.51 (0.26, 0.76), < 0.001	0.002

Abbreviations: PGV = Peak growth velocity.

^†^Adjusted for maternal age, BMI, education, smoking, parity, child’s sex and gestational age at birth. Models with birthweight z-scores as outcome were not adjusted for sex or gestational age. Stratified models were not adjusted for sex.

^‡^Results are derived from the stratified WQS model allowing for sex-specific weights and including the interaction term WQS*sex (p-value_int_).

Metabolites with WQS estimated weights higher than 3.8% (i.e., higher than equal weighting) were considered chemicals of concern (Table 4). The chemicals of concern for lower birthweight z-score were PFOA, Triclosan, HCB, 2OHPH, MCiNP, BPS, PFDA, and MBP, accounting for 74% of the WQS index. The chemicals of concern for a slower infant growth spurt rate were DPP, PFOA, Triclosan, ΣPCBs, MOiNCH, BPF, PFDA, MEP, and 3-PBA, accounting for 79% of the WQS index. For later age at infant PGV, the chemicals of concern were PFOA, BPA, MOiNCH, MEP, ΣPCBs, DPP, Triclosan, and MBzP, accounting for 79% of the WQS index. Results showed significant variations in the WQS weights between boys and girls for each growth parameter. Noticeably, PFOA had higher weights among girls, whereas, Triclosan had higher weights among boys.

**Table 4:** Overall^†^ and sex-specific weights^‡^ in the WQS linear regression analysis between prenatal EDC mixture analysis and children’s growth characteristics, overall and by sex, n = 1,118.

Components of the EDC mixture				Birthweight z-scores				Infant growth spurt rate			Age at infant PGV		
Matrix	Chemical Class	Parent compound	Analyte	Overall		Boys	Girls	Overall	Boys	Girls	Overall	Males	Females
		(if applicable**)**		Weights (%)		Sex-specific weights (%)	Sex-specific weights (%)	Weights (%)	Sex-specific weights (%)	Sex-specific weights (%)	Weights (%)	Sex-specific weights (%)	Sex-specific weights (%)
**Urine**	Phthalates	DEP	MEP	2.9		3.2	3.3	**4.3**	3.4	**6.6**	**7.5**	3.3	**10.9**
		DBP	MBP	**6.1**		1.1	**9.0**	0.1	3.4	0.1	1.0	1.6	0.4
		BBzP	MBzP	2.0		1.9	**7.0**	2.0	**3.8**	0.7	**4.7**	**4.0**	3.1
		DEHP	SumDEHP	2.0		0.8	3.5	3.1	1.6	1.9	1.0	2.2	< 0.1
		DINP	SumDINP	< 0.1		0.9	< 0.1	3.5	3.0	**9.6**	1.3	0.9	0.8
		DiDP/DPHP	MHiDP	0.6		1.1	< 0.1	0.1	1.6	0.8	< 0.1	3.1	0.7
			MCiNP	**9.0**		**4.6**	**9.9**	0.2	**7.4**	**4.3**	1.6	**8.0**	**7.9**
	Plasticizer	DiNCH	MOiNCH	3.8		**10.7**	0.6	**7.5**	**4.1**	**9.5**	**10.9**	**5.3**	**7.8**
		TTP	DPP	0.9		2.6	0.4	**15.7**	**8.9**	**6.7**	**5.7**	3.3	2.0
	Antibacterial		Triclosan	**11.6**		**16.7**	**4.0**	**13.9**	**8.0**	**5.8**	**4.9**	3.2	**7.2**
	Bisphenols		BPA	0.8		1.9	0.9	1.2	3.3	1.7	**11.4**	**3.9**	**9.1**
			BPF	0.1		2.0	0.2	**5.5**	3.5	3.5	2.5	2.9	1.3
			BPS	**7.7**		**9.2**	2.4	0.5	**3.8**	0.6	2.9	3.3	2.1
	PAH		2OHPH	**10.4**		**7.8**	**7.4**	2.1	**4.1**	**4.8**	1.7	**8.1**	**6.5**
	Pesticide	Chlorpyrifos	TCP	0.6		**5.9**	< 0.1	3.0	**5.4**	2.5	3.2	**5.1**	1.2
		Pyrethroids	3-PBA	1.5		1.3	**7.4**	**4.1**	**4.1**	**10.2**	2.9	**5.9**	**7.0**
**Serum**	PFAS		PFOA	**12.6**		**5.8**	**11.2**	**14.1**	3.0	**16.1**	**27.7**	**6.0**	**22.7**
			PFOS	2.1		1.1	**5.5**	0.3	2.6	0.7	< 0.1	2.6	< 0.1
			PFNA	2.1		1.0	**4.8**	1.5	2.4	2.8	1.1	2.8	**4.2**
			PFDA	**6.2**		**4.9**	**5.7**	**5.2**	1.5	**6.5**	0.1	**6.1**	0.3
			PFUnDA	0.6		1.3	0.6	0.9	3.2	0.5	< 0.1	3.2	0.4
			PFHxS	1.0		1.7	1.0	< 0.1	3.3	0.2	0.8	3.7	0.7
**Plasma**	Organo-chlorine pesticide	DDT	HCB	**11.2**		**8.2**	**6.9**	2.0	**4.2**	0.4	0.2	1.0	0.3
			Trans-Nonachlor	1.0		1.7	1.0	< 0.1	2.4	0.4	0.3	3.7	0.7
			DDT/DDE	3.5		1.3	**7.2**	0.9	2.7	0.7	0.3	1.5	0.1
	PCB		SumPCB	< 0.1		1.6	0.2	**8.4**	**5.4**	2.5	**6.4**	**5.4**	2.7

^†^Adjusted for maternal BMI, education, smoking, parity, child’s sex and gestational age at birth. Models with birthweight z-scores as outcome were not adjusted for sex or gestational age. Stratified models were not adjusted for sex.

^‡^ Weights represent the percentage attributable to each component of WQS index, and the sex-specific weights is that percentage calculated within each group (boys or girls). Chemicals with weights > 3.85% are marked in bold.

Sensitivity analyses using single-chemical linear regression models, confirmed that PFOA was associated with lower birthweight z-scores, slower infant growth spurt rate, and later age at PGV (Beta = − 0.349; Beta = − 0.067; Beta = 0.695; respectively) (p-value < 0.05) (Table S2). Also, BPS, PFDA, and HCB were associated with lower birthweight z-scores. However, the other chemicals did not reach significance in the single-chemical model approach.

## Discussion

Prenatal exposure to a one-unit increase in the EDCs WQS-index, was associated with a decrease of 0.11 birthweight z-scores which is similar to the magnitude of effect as maternal smoking during pregnancy has on birthweight z-scores^33^. The chemicals of concern for lower birthweight z-scores were PFOA, Triclosan, HCB, 2OHPH, MCiNP, BPS, PFDA, and MBP. In addition to lower birthweight, our results suggest that exposure to a mixture of EDCs is associated with a slower rate of weight gain and delayed timing of PGV which would shift the infant growth curve towards the right. The chemicals of concern for slower infant growth spurt rate and later age at infant PGV were PFOA, PFDA, Triclosan, ΣPCBs, BPA, BPF, MOiNCH, DPP, MEP, MBzP, and 3-PBA.

Exposure to these chemicals is not unique to pregnant women in Sweden but similar concentrations of phthalates, phenols, and PFASs, have also been found among women in the general population from the US, Mexico and Europe^34–37^. These studies show that most chemicals are detectable in 90–100% of the women, indicating common exposure. Among phthalate and phenols, the urinary concentrations of the DEHP metabolites and BPA in the SELMA study are similar to those reported among women in the US, Mexico and Spain, except for MECPP which was lower^36–38^. Whereas, the reported concentrations of MBzP are higher, and MEP lower in the SELMA study as compared to women in the Netherlands, Spain and Mexico. PFASs concentrations are similar to those reported among women in Denmark but lower than women in the US^34,35^. PFOS and PFOA are the compounds with the highest concentrations among the PFASs analyzed in these studies as in the SELMA study. These concentrations, even though they may vary slightly across populations, are indicative of common exposure among pregnant and nonpregnant women. Consequently, the health effects from exposure to EDC mixtures may be a global matter.

The only previous study that analyzed EDC mixture and birthweight, identified inverse associations between birthweight and PFASs and organophosphate pesticides in a mixture of 53 compounds^29^. Our result overlaps with this mixture analysis in the sense that PFASs are the chemicals driving the association with highest WQS weights in the EDC mixture. In addition to the study on mixture, previous single-component analyses have also found prenatal exposure to PFOA, PFDA, and other PFAS compounds (PFOS, PFNA and PFUnDA) associated with lower birthweight z-score or small-for-gestational age (SGA) birth^19–21,29,39–42^. In terms of Triclosan, there is some evidence from a meta-analysis and two cohorts reporting associations with lower birthweight^22–24^. However, contrasting results of no association have also been found^43,44^. Sex-specific effects have been found with smaller birth size among boys^31^, which is in line with our study showing higher WQS weight for Triclosan in boys.

There is less evidence for the other chemicals of concern associated with lower birthweight z-scores. HCB has been associated with a higher risk of SGA for girls^39^. Similarly, prenatal exposure to PAHs has been associated with smaller birth size and lower weight during the first two years of life^26^. A study in the US found an association between BPS and lower birthweight z-scores but a non-significant trend in the association with MCiNP^25^.

In regards to postnatal growth, our results are in line with Barker and the DOHaD hypothesis which suggests that environmental factors during pregnancy can influence children’s early growth trajectory^9,10^. Exposure to EDC mixtures, beside influencing the growth trajectory, may also interfere with other metabolic processes. Hence, it would be important to further evaluate long-term health effects from a slower infant weight gain in relation to exposure of EDC mixtures.

Only one previous study has evaluated prenatal exposure to a mixture of 27 EDCs and found associations between organochlorine pesticides (i.e. DDE, HCB and PCBs) and increased weight at 7 years of age^30^.

Previous single-compound analyses from the SELMA study showed that exposure to PFOA was associated with lower birthweight^19^ and weight trajectory^32^ only among girls. Our mixtures approach confirmed PFOA was a chemical of concern for the parameters of children’s weight trajectory. In comparison with other studies, evaluating children’s weight at specific time points, we can observe the following associations with PFAS’s. Our longitudinal approach on children’s weight may not be directly comparable but these studies are still informative on the relationship between PFASs and children’s growth. A study in Korea found a relationship specifically with PFNA and lower weight at 2 years of age^46^, Similarly, a study in Denmark found an association between PFOA and decreased weight and body mass index at 5 and 12 months, however, only among boys^21^. Differing results have been found in a Swedish study showing a positive trend between PFOA and BMI z-scores at 4–5 years of age^40^. Besides epidemiological studies, there is emerging evidence that PFOA may interact with sex hormone function, serum levels and receptor functions^47–49^. This may provide some explanation for the associations with growth.

Less information is available on the other chemicals of concern we found for weight trajectory. In regards to PCBs, two studies in the US and Sweden showed that prenatal exposure to PCBs, mainly through contaminated fish or farm products, was related to lower weight of children at 4 and 7 years of age^50,51^. In contrast, other studies have found associations with PCBs and increased weight or body mass index at 3 and 7 years of age^30,52^. Prenatal exposure to BPA has been associated with decreased BMI, body fat, and overweight/obesity among prepubertal girls^53^. In regards to Triclosan, the opposite have been found with higher weight z-scores at 2 years of age^44^. DINCH is a non-phthalate plasticizer used as a replacement for high-molecular phthalates and detected in 99% of the samples collected in the SELMA study^54^. There is limited information on the effect of prenatal exposure to DINCH and children’s postnatal growth. In one study, exposure to DINCH induced preadypocytes to accumulate lipids and differentiate into mature adipocytes by activating peroxisome proliferator activated receptor (PPAR)-alpha pathway, similar to other phthalates^55,56^. In contrast, another study reported no obesogenic effect associated with prenatal exposure to DINCH on body weight and other cardiometabolic markers (e.g. lipids) in rat pups^57^. DINCH has also shown to cause cytotoxicity in kidney cells and DNA damage to liver cells, indicating that it is hazardous to human cells^58^. Hence, exposure to DINCH has been associated with negative effects on human cells but there is not a clear consensus of the effect on growth and obesity.

This study has strengths worth highlighting. The data was collected in a large ongoing longitudinal study which have followed mother-infant pairs from early pregnancy. Most previous studies evaluating prenatal exposure to EDCs and children’s growth have looked at specific time points in early infancy, whereas we were able to consider the shape of the curve for children’s weight trajectory. This adds valuable information on the effect of EDCs on children’s weight gain over time. Some advantages of our modelling approach for estimating growth, as compared to other more complex models^59–62^, is a simple computational approach with the ability to derive further growth metrics (e.g. growth acceleration, peak growth velocity), and the estimation of individual growth metrics which provides utility of them in later regression analysis. On the other hand, it requires frequent measurements over time, as greater amount of measures improves the estimation of the growth metrics and the selection of the nonlinear model^32^.

Our study included a mixture of EDCs including phthalates, phenols, PFAS, PCBs, organochloride pesticides and other short-lived chemicals as PAHs. In order to define the feasibility of a mixture-centered approach for chemical risk assessment, we focused in this study on 26 chemicals belonging to different chemical groups. The rationale for this selection, which represents only a portion of all the chemicals we currently are exposed to, was based on established evidence, both of their endocrine interfering properties of importance for metabolism and growth, their ubiquitous occurrence, and their association to metabolism and growth (from single compound studies). We chose WQS regression to analyze mixtures in our analyses due to several reasons. Considering that exposures to EDCs generally occur in complicated mixtures, the WQS regression allowed for estimation of the overall mixture effect on children’s weight providing simplicity of interpretation of the WQS index as well as the weights. Our approach of using WQS regression also permitted the identification of those chemicals driving the associations with each growth parameter, as well as allowing for different slopes and calculate sex-specific weights. In terms of handling correlated exposures, as in the case of EDC metabolites, WQS regression has shown to perform with good sensitivity and specificity in several studies relevant to environmental exposures, including studies handling correlated high-dimensional data^65–72^. In comparison with other shrinkage methods (e.g. lasso, elastic net), WQS regression results in similar or improved identification of the chemicals of concern^66^. As compared to other methods that analyze mixtures, WQS regression is preferred in hypothesis driven analyses to evaluate association in a certain direction with the health outcome. Unsupervised approaches such as principal component analysis (PCA) or factor analysis (FA) may be preferable when there is no hypothesis a priori for the direction of the association.

When comparing results from the single-chemical modelling approach, many of the chemicals did not reach significance in the association with the growth parameters. This may be because mixture modelling approaches may identify chemicals which would not be deducible from a single-chemical approach, due to the “cocktail effect” (e.g. additive, synergistic effects)^63,64^.

Our results should be interpreted in light of the following limitations. The modelling of children’s weight provided an estimation of a PGV at approximately three months of age which is later than what is usually described^73^. More frequent measures during the first weeks of life in the model might have been more sensitive to earlier growth spurts. Urine samples were only collected once during pregnancy. Therefore, we are not able to identify variations in exposure over time during pregnancy in particular for non-persistent EDCs. Previous biomonitoring studies on urinary concentrations of non-persistent EDCs (e.g. phthalates, phenols) have shown that within subject variability of exposure measured in single urine-spot samples shows a somewhat stable variability over time but may vary across pregnancy^74,75^. Hence, we may assume that for most women the urinary concentrations represents daily exposure during early pregnancy. In order to reduce misclassification, urine collection was standardized by using only first-morning voids which are more reliable^76,77^. However, any potential misclassification should be nondifferential with respect to the outcome and therefore we expect bias towards the null. Some of the limitations of using WQS regression is that it assumes there is no interaction between exposure, as well as constant change in risk between the quantiles. To assess the linearity assumption, LOESS plots between the WQS index and the covariate adjusted outcome are reviewed and quadratic terms can be added. In our analysis, our main goal was to estimate the overall mixture effect whereas other methods may be preferable for the evaluation of interactions between single exposures in a mixture (e.g. Bayesian kernel machine regression (BKMR))^78^. Although, the use of quantiles reduces the influence of outliers of exposure it also reduces the information of the full range of exposure^66^. Our results may also have limited generalizability as we did not split the data into training and validation set when performing WQS regression model due to our sample size which did not allow for stable estimation of weights.

Consistent with Barker and the DOHaD hypothesis, our results demonstrate that prenatal exposure to environmental factors influence early growth, specifically a change in growth curve trajectories. This may be relevant in regards to long-term health effects and metabolic diseases. Future investigations on the metabolic health impacts of prenatal EDC mixture exposure in infancy through adulthood are critical areas of research.

## Conclusions

Our results shows that prenatal exposure to EDC mixture have an impact on pre- and postnatal growth leading to lower birthweight z-scores and slower infancy weight gain. In a mixture of EDCs, we found evidence that both persistent and short-lived chemicals are of concern for children’s growth. It may be especially important to limit exposure to EDCs for pregnant women and children as they represent life stages sensitive for growth and development.

## Methods

### Study population

This study is based on data from the SELMA study; an ongoing prospective study which recruited 2,582 pregnant women in 2007–2010 at approximately 10 weeks of gestation. The recruitment protocol has been described previously in more detail^79^. For this analysis we selected, 1,118 mother–child pairs with data on 26 suspect EDCs, child’s weight, and complete data on the selected covariates. Out of 2,582 pregnant women recruited into the SELMA study, a total of 1,549 mother–child pairs had information on children’s weight measurements (birth until 5.5 years of age), and 1,323 had measured EDCs concentrations from urine and blood samples during pregnancy. From those 1,323 mother–child pairs, a total of 205 had missing values either on maternal education (n = 185) or maternal BMI (n = 52), and therefore excluded from the analysis. This resulted in a final sample of 1,118 mother–child pairs. Children excluded due to any missing data (n = 783) had larger birthweight (3.7 kg vs. 3.6 kg) and their mothers were more likely to have lower education level (high school: 43.3% vs. 36.0%), smoke (9.9% vs. 5.5%), and multiparous (60.9% vs. 51.3%) (Table S3). The study was performed in accordance with the Declaration of Helsinki. All participating women signed informed consent for theirs and their children’s participation, and the study has been approved by the Regional Ethical Review Board in Uppsala, Sweden (2007-05-02, Dnr: 2007/062 and Dnr: 2015/177).

### Sample collection and measurement of EDCs

Women provided blood and first morning void urine samples during their 1^st^ trimester of pregnancy during the enrollment visit at a prenatal care center. All samples were kept frozen until analysis (− 80ºC for serum and plasma, and − 20ºC for urine)^79^. A total of 54 analytes with either proven or suspected endocrine disrupting properties were analyzed. From these compounds, we selected 41 metabolites with detectable values in at least 75% of the samples. After summation there were 26 metabolites and compounds included in the analysis (Table S1)^80^.

Urinary metabolites of nonpersistent chemicals with short biological half-life were analyzed using liquid chromatography coupled to a triple quadrupole mass spectrometer (LC–MS/MS) according to a method presented by Gyllenhammar et al. 2017^81^. Serum was analyzed for PFAS and cotinine using LC/MS/MS according to Lindh et al. 2012^82^. The laboratory is part of Erlangen Round Robin inter-laboratory control program and has qualified as HBM4EU laboratory for several compounds. Plasma samples were analyzed for persistent organic pollutants using gas chromatography–MS/MS (GC–MS/MS) according to Koponen et al., 2013^83^. Spearman correlations coefficients between these chemicals have been reported previously and ranged between − 0.16 and 0.75, with urinary creatinine-adjusted chemicals being low to moderately correlated and persistent chemicals highly correlated^80^. More detailed descriptions of the analytical methods for blood and urine samples has been described previously^81,82,84^.

We calculated summed variables for certain metabolites. The sum of DEHP and DINP metabolites were calculated on a molar basis. We also summed DDT with its metabolite DDE, and all the PCB congeners for total exposure variables. For the metabolites measured in plasma, values below the level of detection (LOD) were replaced by the value of LOD/√(2), whereas for serum and urinary metabolites we used machine read values^80^. All urinary metabolites were creatinine adjusted in units of nmol per mol creatinine to adjust for urine dilution in the regression models. The full name of the 41 metabolites as well as the summed variables are listed in Table S1.

### Anthropometric measures and covariates

Children’s weight was measured at birth and after birth up to 15 times during routine health care visits at a Child Health Center (CHC) scheduled at 2 weeks of age, and at 2, 3, 4, 5, 6, 8, 10, 12, 18, 30, 36, 48, and 66 months of age^32^. Birthweight z-scores were calculated based on the Swedish national growth reference^85^. Sociodemographic information was collected through self-administered questionnaires, whereas maternal age, weight, parity, child’s gestational age at birth (based on ulstrasound examinations) and sex was collected through the Swedish medical birth registry. Smoking status of active smokers was determined based on cotinine levels above 15 (ug/dl), and on self-reported status if cotinine values were missing. Covariates were selected based on previous literature or statistical significance in the WQS regression models (p-value < 0.05)^8,34–38,86,87^.

### Statistical analyses

**Figure 1:**
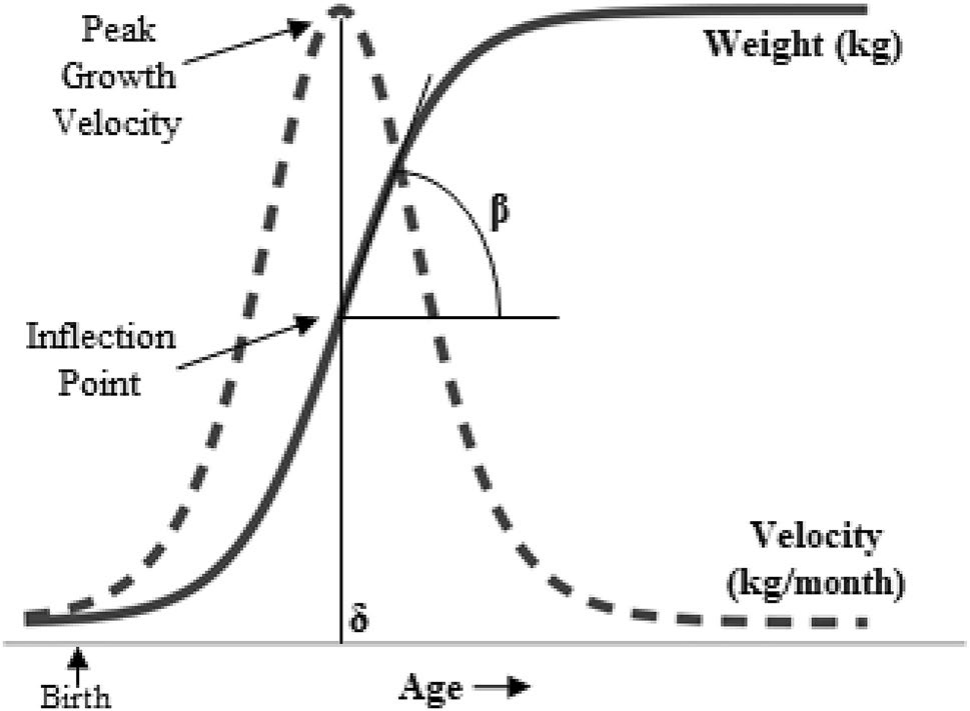
Double-logistic growth model to predict weight trajectories. The double-logistic growth model is in this analysis applied to predict children’s weight trajectories from birth to 5.5 years of age. The infant period is captured by the first of two logistic functions. This first function is exponential with weight (kg) increasing with age (months) and undertakes an inflection point where the rate constant is determined by the slope of the tangent line (β). This slope is in this analysis labeled as the infant growth spurt rate (kg/month). Growth velocity is the first derivative of the logistic growth model, and the peak growth velocity (PGV) is reached at age = δ. (Adapted from Tanner et al., 2020).

Descriptive statistics were used to summarize central tendency measures. Our method of analyzing children’s weight trajectory is built on previously published results from the SELMA study^32^. In brief, a double-logistic growth model was used to model each child’s weight trajectory from birth to 5.5 years of age including in average 11.6 (SD = 1.9) measures of weight per child (8 to 15 measures of weight). The double-logistic model assumes a sigmoidal shape in two sequential growth periods and provides several parameters which are indicative of the shape of the weight trajectory (Fig. 1). For this analysis, we selected the following parameters: infant growth spurt rate (kg/month) and age at peak growth velocity (PGV) (months) from the first growth period. We log_10_ transformed the infant growth spurt rate to approximate a normal distribution. The infant growth spurt rate is the tangent at the inflection point of the curve and occurs at the same age as the PGV. Both parameters provide similar information in terms of the child’s weight trajectory. However, because we are interested in the shape of the curve we chose to analyze only the infant growth spurt rate.

To evaluate the association between mixtures of EDCs and the weight trajectory parameters, we used weighted quantile sum (WQS) regression which derives a weighted index estimating the mixture effect associated with each parameter, and also identifies chemicals of concern in the mixture through estimated weights^66,69^. The WQS regression has the following equation:

$$g\left(\mu \right)= {\beta }_{0}+{\beta }_{1}\left(\sum_{i=1}^{c}{w}_{i}{q}_{i}\right)+{z}^{^{\prime}}\varphi$$

where g() is the link function (in this case generalized linear model), µ is the mean of the outcome, q_i_ is the quantile of the i^th^ component (here, deciles), w_i_ is the weight associated with the i^th^ component, ${z}^{^{\prime}}$ is the vector of covariates and $\varphi$ is the vector of parameters associated with the covariates. The term $\left(\sum_{i=1}^{c}{w}_{i}{q}_{i}\right)$ represents the index that weighs and sums the components included in the mixture. The weights associated with each component in the mixture are estimated as the average from 100 bootstrap samples. The chemicals with higher weights account for higher contribution to the weighted index. We did not split the data into training and validation set as the sample size did not allow for stable weight estimates. Each of the weighted indices were then assessed with the respective outcome using linear regression models and adjusting for covariates. For each outcome, it is possible to derive both a positively and negatively associated index. By estimating one index at the time it focuses the inference, and thereby reducing some of the ill conditioning due to the complex correlation pattern. For birthweight z-scores we modeled an index associated with lower birthweight z-scores based on results from previous literature on EDCs and birthweight. For the other outcomes we ran WQS regression deriving indices in both directions and report the index where we found a significant association with the respective outcome. We also ran models with the interaction term WQS*sex and a stratified WQS regression with sex-specific weights^88^. These extensions to WQS regression have the advantage of estimating the weights in the presence of the interaction term, and the stratified model with an interaction term permits a different regression coefficient for each level of the strata with strata-specific weights, in this case by sex. If the WQS index in the stratified model is significant, then the sex-specific weights are different between boys and girls and indicating different ranks and magnitudes of the components in the mixture. The WQS regression models were conducted using the R package “gWQS: generalized weighted quantile sum regression” version 2.0^89^. All the models were adjusted by covariates selected a priori based on current literature and statistical significance (p-value < 0.05): maternal age, BMI, education, smoking, parity, child’s sex and gestational age at birth. The models with birthweight z-scores as outcome was not adjusted by sex or gestational age as this is already considered in the calculation of the z-scores. Also, the stratified models were not adjusted for sex.

We ran sensitivity analysis with single-chemical regression models with each of the 26 metabolites and the growth parameters; birthweight z-scores, infant growth spurt rate and age at PGV. All the analyses were performed using the statistical software R version 3.5.2.

## Supplementary Information

Supplementary Information.

## Data Availability

According to the Ethical Review Board decision and obtained personal consent, data on participating children or their mothers can not be made freely available. This since they constitute clinical data subject to secrecy in accordance with the Swedish Public Access to Information and Secrecy Act [OSL 2009:400]. Unique combinations of clinical data could make a study participant identifiable, and consequently a review of secrecy may result in restrictions regarding data availability.

## Abbreviations

EDC: Endocrine disrupting chemicals
PGV: Peak growth velocity
2OHPH: 2-Hydroxyphenanthrene
BPA: Bisphenol A
BPF: Bisphenol F
BPS: Bisphenol S
DEHP: Di-(2-ethylhexyl) phthalate
DDT: Dichlorodiphenyltrichloroethane
DINP: Diisononyl phthalate
DOHaD: Developmental origins of health and disease
DPP: Diphenylphosphate
HCB: Hexachlorobenzene
MBP: Monobutyl phthalate
MBzP: Monobenzyl phthalate
MCiNP: Monocarboxyisononyl phthalate
MEP: Monoethyl phthalate
MOiNCH: 2–4-Methyl-7-oxyooctyl-oxycarbonyl-cyclohexane carboxylic acid
3-PBA: 3-Phenoxybenzoic acid
PCB: Polychlorinated biphenyl
PFAS: Perfluoroalkyl substance
PFDA: Perfluorodecanoic acid
PFNA: Perfluorononanoic acid
PFOA: Perfluorooctanoic acid
PFOS: Perfluorooctane sulfonate
PFUnDA: Perfluoroundecanoic acid
