# Supplementary material for: Prenatal exposures to mixtures of endocrine disrupting chemicals and children’s weight trajectory up to age 5.5 in the SELMA study
Source: Sci Rep. 2021 May 26;11:11036. doi: 10.1038/s41598-021-89846-5 (PMC8155069; doi:10.1038/s41598-021-89846-5)
Supplement: Supplementary file 1 — Supplementary Information. [file 41598_2021_89846_MOESM1_ESM.docx]

**Supplementary material:**

**Prenatal exposures to mixtures of endocrine disrupting chemicals and children’s weight trajectory up to age 5.5 in the SELMA study**

Katherine Svensson, MS^1^, Eva Tanner, PhD^2^, Chris Gennings, PhD^2^, Christian Lindh, PhD^3^, Hannu Kiviranta, PhD^4^, Sverre Wikström, MD, PhD^5^, *Carl-Gustaf Bornehag, PhD^1,2^

^1^ Department of Health Sciences, Karlstad University, Karlstad, Sweden

^2^ Department of Environmental Medicine and Public Health, Icahn School of Medicine at Mount Sinai, New York, NY, US

^3^ Division of Occupational and Environmental Medicine, Lund University, Lund, Sweden

^4^ Environmental Health Unit, Finnish Institute for Health and Welfare, Kuopio, Finland

^5^ School of Medical Sciences, Örebro University, Örebro, Sweden

**Corresponding author:**

*Carl-Gustaf Bornehag, PhD

telephone number: +46 70 586 6565

email: carl-gustaf.bornehag@kau.se

address: Department of Health Sciences, Karlstad University, Universitetsgatan 2, 651 88 Karlstad, Sweden

**Table S1. Concentrations of metabolites (ng/mL) in prenatal urine (not creatine adjusted) and blood samples, n=1,118**

| **Components of the EDC mixture** | | | | | **Overall**  **(n=1,118)** | | |
| --- | --- | --- | --- | --- | --- | --- | --- |
| **Matrix** | **Chemical Type** | **Parent Compound** | **Analyte** | **Abbreviation** | **LOD/LOQ^a^** | **% ≥ LOD** | **GM (GSD)** |
|  |  | **(*if applicable*)** |  |  |  |  |  |
| **Urine** | Phthalates | DEP | monoethyl phthalate | MEP | 0.010 | 100 | 67.5 (2.9) |
|  |  | DBP | monobutyl phthalate | MBP | 0.100 | 100 | 67.4 (2.2) |
|  |  | BBzP | monobenzyl phthalate | MBzP | 0.040 | 100 | 15.6 (2.9) |
|  |  | DEHP | mono(2-ethylhexyl) phthalate | MEHP | 0.100 | 100 | - |
|  |  |  | mono(2-ethyl-5-hydroxyhexyl) phthalate | MEHHP | 0.020 | 100 | - |
|  |  |  | mono(2-ethyl-5-oxohexyl) phthalate | MEOHP | 0.030 | 100 | - |
|  |  |  | mono(2-ethyl-5-carboxypentyl) phthalate | MECPP | 0.020 | 100 | - |
|  |  |  | di-(2-ethylhexyl) phthalate | ΣDEHP^b^ | - | - | 63.8 (2.4) |
|  |  | DINP | mono(hydroxy-iso-nonyl) phthalate | MHiNP | 0.020 | 100 | - |
|  |  |  | mono(oxo-iso-nonyl) phthalate | MOiNP | 0.010 | 100 | - |
|  |  |  | mono(carboxy-iso-octyl) phthalate | MCiOP | 0.020 | 100 | - |
|  |  |  | diisononyl phthalate | ΣDINP^c^ | - | - | 25.6 (3.0) |
|  |  | DiDP/DPHP | monohydroxyisodecyl phthalate | MHiDP | 0.031 | 100 | 1.23 (2.8) |
|  |  |  | monocarboxyisononyl phthalate | MCiNP | 0.031 | 99.9 | 0.66 (2.4) |
|  | Plasticizer | DiNCH | 2-4-methyl-7-oxyooctyl-oxycarbonyl-cyclohexane carboxylic acid | MOiNCH | 0.023 | 99.0 | 0.30 (4.0) |
|  |  | TTP | diphenylphosphate | DPP | 0.042 | 100 | 1.38 (2.6) |
|  | Antibacterial |  | 2,4,4′-trichloro-2′-hydroxydiphenyl ether | Triclosan | 0.100 | 92.4 | 1.34 (10.1) |
|  | Bisphenols |  | bisphenol A | BPA | 0.050 | 100 | 1.48 (2.4) |
|  |  |  | 4,4-bisphenol F | BPF | 0.024 | 90.3 | 0.15 (5.2) |
|  |  |  | bisphenol S | BPS | 0.009 | 97.5 | 0.07 (2.9) |
|  | Polycyclic aromatic hydrocarbon (PAH) |  | 2-hydroxyphenanthrene | 2OHPH | 0.003 | 100 | 0.20 (2.3) |
|  | Pesticide | Chlorpyrifos | 3,5,6-trichloro-2-pyridinol | TCP | 0.035 | 100 | 1.30 (2.5) |
|  |  | Pyrethroids | 3-phenoxybenzoic acid | 3-PBA | 0.017 | 99.0 | 0.16 (2.8) |
| **Serum** | PFAS |  | perfluorooctanoic acid | PFOA | 0.020 | 100 | 1.63 (1.7) |
|  |  |  | perfluorooctane sulfonate | PFOS | 0.060 | 100 | 5.49 (1.7) |
|  |  |  | perfluorononanoic acid | PFNA | 0.010 | 100 | 0.55 (1.7) |
|  |  |  | perfluorodecanoic acid | PFDA | 0.020 | 100 | 0.26 (1.6) |
|  |  |  | perfluoroundecanoic acid | PFUnDA | 0.020 | 99.7 | 0.22 (1.9) |
|  |  |  | perfluorohexanesulfonic acid | PFHxS | 0.030 | 100 | 1.31 (1.8) |
| **Plasma** | Organochlorine pesticide |  | hexachlorobenzene | HCB | 0.005 | 100 | 0.05 (1.4) |
|  |  |  | trans-nonachlor | Nonachlor | 0.005 | 77.5 | 0.01 (1.8) |
|  |  | DDT | dichlorodiphenyltrichloroethane | DDT | 0.015 | 7.5 |  |
|  |  |  | dichlorodiphenyldichloroethylene | DDE | 0.040 | 99.5 | - |
|  |  |  | total dichlorodiphenyltrichloroethane | ΣDDT/DDE^d^ | - | - | 0.20 (2.0) |
|  | PCB |  | polychlorinated biphenyl 74 | PCB 74 | 0.005 | 72.4 | - |
|  |  |  | polychlorinated biphenyl 99 | PCB 99 | 0.005 | 80.7 | - |
|  |  |  | polychlorinated biphenyl 118 | PCB 118 | 0.005 | 98.7 | - |
|  |  |  | polychlorinated biphenyl 138 | PCB 138 | 0.005 | 100 | - |
|  |  |  | polychlorinated biphenyl 153 | PCB 153 | 0.005 | 100 | - |
|  |  |  | polychlorinated biphenyl 156 | PCB 156 | 0.005 | 89.8 | - |
|  |  |  | polychlorinated biphenyl 170 | PCB 170 | 0.005 | 100 | - |
|  |  |  | polychlorinated biphenyl 180 | PCB 180 | 0.005 | 100 | - |
|  |  |  | polychlorinated biphenyl 183 | PCB 183 | 0.005 | 76.5 | - |
|  |  |  | polychlorinated biphenyl 187 | PCB 187 | 0.005 | 97.1 | - |
|  |  |  | total polychlorinated biphenyls | ΣPCB^e^ | - | - | 0.36 (1.7) |

Abbreviations: GM = Geometric mean, GSD = Geometric standard deviation, LOD = limit of detection, LOQ = limit of quantification.

Notes: Values<LOD retained the machine read value for urine and serum compounds, values<LOQ were substituted with LOQ/ 2 for plasma compounds.

^a^ LOD reported for all urine and serum compounds, LOQ reported for plasma compounds.

^b^ Molar sum of metabolites: mono-2-ethylhexyl, mono(2-ethyl-5-hydroxyhexyl), mono(2-ethyl-5-oxohexyl), and mono(2-ethyl-5-carboxypentyl) phthalates.

^c^ Molar sum of metabolites: mono(hydroxyisononyl), mono(oxoisononyl), and mono(carboxyisooctyl) phthalates.

^d^ Sum of DDT and its metabolite dichlorodiphenyldichloroethylene.

^e^ Sum of PCB congeners 74, 99, 118, 138, 153, 156, 170, 180, 183, 187.

**Table S2. Adjusted^†^ associations from linear regressions between each creatinine-adjusted log(metabolite) and children’s growth parameter, n=1,118**

| **Components of the EDC mixture** | | | |  | **Birthweight**  **z-scores** | **Log (Infant slope) (kg/months)** | **Age at PGV (months)** |
| --- | --- | --- | --- | --- | --- | --- | --- |
| **Matrix** | **Chemical Class** | **Parent Compound** | **Analyte** |  |  |  |  |
|  |  | **(*if applicable*)** |  |  | **Beta, p-value** | **Beta, p-value** | **Beta, p-value** |
| **Urine** | Phthalates | DEP | MEP |  | -0.017, 0.804 | -0.005, 0.737 | 0.139, 0.218 |
|  |  | DBP | MBP |  | -0.111, 0.328 | 0.014, 0.560 | 0.125, 0.504 |
|  |  | BBzP | MBzP |  | -0.038, 0.628 | 0.000, 0.982 | 0.074, 0.567 |
|  |  | DEHP | SumDEHP |  | -0.085, 0.351 | -0.013, 0.480 | 0.075, 0.618 |
|  |  | DINP | SumDINP |  | 0.045, 0.492 | -0.009, 0.513 | 0.096, 0.374 |
|  |  | DiDP/DPHP | MHiDP |  | -0.068, 0.361 | 0.004, 0.810 | -0.012, 0.925 |
|  |  |  | MCiNP |  | -0.111, 0.189 | 0.011, 0.537 | -0.044, 0.754 |
|  | Plasticizer | DiNCH | MOiNCH |  | -0.007, 0.903 | -0.005, 0.614 | 0.083, 0.332 |
|  |  | TTP | DPP |  | -0.024, 0.767 | -0.026, 0.118 | 0.150, 0.264 |
|  | Antibacterial |  | Triclosan |  | -0.037, 0.214 | -0.006, 0.295 | 0.024, 0.626 |
|  | Bisphenols |  | BPA |  | -0.061, 0.492 | -0.005, 0.788 | 0.251, 0.086 |
|  |  |  | BPF |  | 0.031, 0.471 | -0.008, 0.350 | 0.031, 0.663 |
|  |  |  | BPS |  | **-0.149, 0.032** | 0.011, 0.442 | 0.071, 0.534 |
|  | PAH |  | 2OHPH |  | -0.076, 0.416 | 0.013, 0.508 | -0.086, 0.573 |
|  | Pesticide | Chlorpyrifos | TCP |  | 0.066, 0.402 | 0.006, 0.720 | -0.044, 0.736 |
|  |  | Pyrethroids | 3-PBA |  | 0.004, 0.956 | -0.015, 0.327 | 0.082, 0.510 |
| **Serum** | PFAS |  | PFOA |  | **-0.349, 0.013** | **-0.067, 0.020** | **0.695, 0.003** |
|  |  |  | PFOS |  | -0.181, 0.181 | -0.017, 0.540 | -0.042, 0.850 |
|  |  |  | PFNA |  | -0.217, 0.108 | -0.039, 0.160 | 0.365, 0.101 |
|  |  |  | PFDA |  | **-0.286, 0.048** | -0.048, 0.101 | 0.185, 0.439 |
|  |  |  | PFUnDA |  | -0.083, 0.468 | -0.005, 0.825 | -0.018, 0.925 |
|  |  |  | PFHxS |  | 0.051, 0.663 | 0.032, 0.181 | -0.046, 0.815 |
| **Plasma** | Organo-chlorine pesticide |  | HCB |  | **-0.537, 0.018** | -0.027, 0.564 | -0.058, 0.877 |
|  |  |  | Trans-Nonachlor |  | -0.160, 0.228 | -0.003, 0.904 | -0.016, 0.942 |
|  |  | DDT | DDT/DDE |  | -0.173, 0.091 | 0.006, 0.787 | -0.105, 0.533 |
|  | PCB |  | SumPCB |  | -0.021, 0.895 | -0.025, 0.439 | 0.130, 0.621 |

^†^Adjusted for maternal BMI, education, smoking, parity, child’s sex and gestational age at birth.

**Table S3. Sociodemographic characteristics of the study population as compared to excluded participants**

|  | **Non-sample**  **(n=783)** | **Study Sample**  **(n=1,118)** | **P-value*^*^*** |
| --- | --- | --- | --- |
| **Continuous variables** | **Mean (SD)** | **Mean (SD)** |  |
| Maternal age (years) | 31.1 (5.2) | 30.9 (4.7) | 0.274 |
| Maternal BMI (kg/m^2^) | 25.0 (4.8) | 24.7 (4.3) | 0.193 |
| Infant’s gestational age at birth (weeks) | 39.6 (1.9) | 39.5 (1.7) | 0.260 |
| Birthweight (kg) | 3.679 (0.587) | 3.610 (0.543) | 0.010 |
|  |  |  |  |
| **Categorical variables** | **n (%)** | **n (%)** |  |
| *Maternal Education* |  |  |  |
| Primary school or high school | 280 (43.3) | 402 (36.0) |  |
| College or higher | 366 (56.7) | 716 (64.0) | 0.003 |
| *Smoking* |  |  |  |
| Non-smoker | 643 (90.1) | 1,056 (94.5) |  |
| Smoker | 71 (9.9) | 62 (5.5) | <0.001 |
| *Parity* |  |  |  |
| Nulliparous | 306 (39.1) | 544 (48.7) |  |
| Multiparous | 477 (60.9) | 574 (51.3) | <0.001 |

^*^ P-value, based on Student T-test for continuous variables and Chi-square test for categorical variables.
